# Immunological analysis of a *Lactococcus lactis-*based DNA vaccine expressing HIV gp120

**DOI:** 10.1186/1479-0556-5-3

**Published:** 2007-01-29

**Authors:** Gregers J Gram, Anders Fomsgaard, Mette Thorn, Søren M Madsen, Jacob Glenting

**Affiliations:** 1 Department of Virology, State Serum Institute, Artillerivej 5, DK-2300 Copenhagen, Denmark; 2 Vaccine Technology, Bioneer A/S, Kogle Alle 2, DK-2970Hørsholm, Denmark

## Abstract

For reasons of efficiency *Escherichia coli *is used today as the microbial factory for production of plasmid DNA vaccines. To avoid hazardous antibiotic resistance genes and endotoxins from plasmid systems used nowadays, we have developed a system based on the food-grade *Lactococcus lactis *and a plasmid without antibiotic resistance genes. We compared the *L. lactis *system to a traditional one in *E. coli *using identical vaccine constructs encoding the gp120 of HIV-1. Transfection studies showed comparable gp120 expression levels using both vector systems. Intramuscular immunization of mice with *L. lactis *vectors developed comparable gp120 antibody titers as mice receiving *E. coli *vectors. In contrast, the induction of the cytolytic response was lower using the *L. lactis *vector. Inclusion of CpG motifs in the plasmids increased T-cell activation more when the *E. coli *rather than the *L. lactis *vector was used. This could be due to the different DNA content of the vector backbones. Interestingly, stimulation of splenocytes showed higher adjuvant effect of the *L. lactis *plasmid. The study suggests the developed *L. lactis *plasmid system as new alternative DNA vaccine system with improved safety features. The different immune inducing properties using similar gene expression units, but different vector backbones and production hosts give information of the adjuvant role of the silent plasmid backbone. The results also show that correlation between the *in vitro *adjuvanticity of plasmid DNA and its capacity to induce cellular and humoral immune responses in mice is not straight forward.

## Background

Genetic immunization or DNA vaccination has initiated a new era of vaccine research. The technology involves the inoculation of plasmid DNA into a living host to elicit an immune response to a protein encoded on the plasmid [1]. The potential advantages of DNA vaccines include the induction of cellular and humoral immune responses, flexible genetic design, lack of infection risk, stability of reagents, and the relatively low cost of production in a microbial host. These advantages are being exploited for infections like HIV where traditional vaccines have proved unsuccessful [2-5]. The advantages of DNA vaccines have lead to extensive research primarily focused on the immune responses induced against a variety of antigens but less on the tools required for the microbial production of plasmids. Plasmid DNA used for DNA vaccinations have two general features reflecting its dual functionality: the unit responsible for propagation in microbial cells called the plasmid backbone, and the unit that expresses the vaccine gene in the transfected tissue. For reasons of efficiency, *Escherichia coli*, with its concomitant benefits and drawbacks, has been the production host of choice. The benefits include a high DNA yield and well-established procedures for down-stream processing of the plasmid [6]. However, as a gram-negative bacterium, *E. coli *contains highly immunogenic endotoxins, or lipopolysaccharides (LPS), in its outer membrane. Because of the net negative charge of both LPS and DNA, these molecules may be co-purified by the ion exchange principle used in the purification of plasmid DNA [7], although commercial kits do exist that can exclude LPS. On the other hand, the use of gram-positive hosts, none of which produce LPS, eliminate this dependency on the absolute efficiency of LPS-removing kits. In addition, the *E. coli *vaccine plasmids are typically maintained during growth by plasmid-encoded antibiotic resistance and addition of antibiotics to the growth medium. Thus, antibiotics may be contaminants in the purified DNA vaccines with the potential of inducing allergic responses in disposed individuals [8]. Furthermore, there is much scientific and regulatory focus on the use of antibiotic resistance genes. The concern is that the plasmid may transform the patient's microflora and spread resistance genes [9]. We have developed a host vector system for the production of plasmid DNA based on *Lactococcus lactis *with improved safety properties concerning the microbial production host and the genetic content of the plasmid [10]. As host *L. lactis *is attractive for production of vaccines due to its food grade status. The developed plasmid is free of antibiotic resistance genes and is based entirely on *L. lactis *genes; the minimal theta type plasmid replicon [11] and the *hom-thrB *operon [12] encoding the homoserine dehydrogenase and homoserine kinase catalysing two steps in the biosynthesis of threonine. The use of this auxotrophic marker is based on complementation of a *L. lactis *host strain containing an internal deletion in the *hom-thrB *operon on the chromosome. Thus plasmid complementation relieves the strains requirement for threonine. Other food grade genetic markers have been developed in *L. lactis *to meet the high safety demands of optimised and recombinant starter cultures for use in the manufacture of dairy products. An example is the use of suppressible pyrimidine auxotrophs where suppressor tRNA is encoded by the plasmid and allows read through of the *pyrF *gene containing an amber mutation [13]. A selection marker based on lactose utilization has also been developed [14]. Both food-grade genetic markers allow selection in milk containing both lactose and low amount of pyrimidines. However, application of non-antibiotic-based genetic markers in *L. lactis *for plasmid DNA production is new. We have investigated the use of the new *L. lactis*-based plasmid as a potential HIV-1 DNA vaccine by including a eukaryote expression unit encoding the gp120 surface molecule from the primary CCR-5-trophic HIV-1_BX08 _[5,15]. Although naked DNA vaccines may be very helpful in priming the humoral and cellular immune system for subsequent boosting with more immunogenic agents like recombinant virus or proteins [3,15], the relatively low potency of the DNA vaccines themselves is problematic. The lack of immune stimulatory endotoxins or immune stimulatory sequences (ISS) like CpG motifs may influence the potency of the DNA vaccine preparation. Indeed, DNA itself acts as adjuvant [16-19], which depends on the content of ISS CpG motifs and the dose given. In this respect it may be of importance that *L. lactis *genes are relatively AT rich compared to the more GC rich *E. coli *genes and thus may contain fewer putative ISS.

The goal of this study was to develop an alternative host vector system based on *L. lactis *and compare it to a traditional one using same vaccine cassette. The developed system shows potential as a new plasmid production system, but gives also information about the adjuvant role of the silent plasmid backbone in DNA vaccination.

## Methods

### Construction of *E. coli *and *L. lactis *based expression vectors

**Figure 1:**
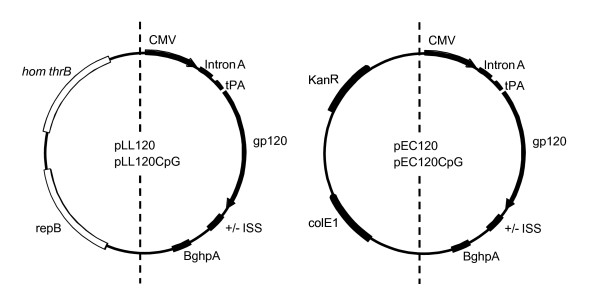
**The genetic anatomy of vaccine plasmids**. Vaccine plasmids from *L. lactis *(pLL120) and *E. coli *(pEC120) with CpG motifs. The plasmids contain a similar expression cassette which carries the CMV promoter and the gp120 gene fused to the signal sequence tPA, and a transcription terminator (BGHpA). Intron A is included for efficient splicing. The pLL120 vector backbone contains the minimal theta type plasmid replicon encoding the repB protein and the *hom-thrB *genetic marker encoding two threonine biosynthetic enzymes homoserine dehydrogenase and homoserine kinase. The pEC120 vector backbone contains the colE1 origin of replication and the kanamycin resistance (KanR) genetic marker.

The *E. coli *expression plasmid WRG7079 (PowderJect Inc., Madison, WI, USA) contains the kanamycin resistance gene, a ColE1-based origin of replication and a eukaryotic expression unit harboring the cytomegalo virus (CMV) promoter and enhancer region, Intron A, the tissue plasminogen activator (tPA) secretion signal, a polylinker, and the polyadenylation signal from bovine growth hormone (BGHpA). The synthetic gp120 gene with human codons from the primary, CCR5-tropic, clade B HIV-1_BX08 _has been cloned into WRG7079 [5] and is named pEC120 (6.2 kb). A variant of pEC120 containing ISS CpG motifs was constructed. An 80 bp CpG cassette (AT**CG**ACTCT**CG**AG**CG**TTCTAT**CG**ACTCT**CG**AG**CG**TTCTCACTCT**CG**AG**CG**TTCT**CG**CTAGA**CG**TTAG**CG**TTCAA**CG**TTGA) containing 12 CpGs (bold) including human and mouse ISS motifs [20-23], was introduced between the stop codon of the gp120 gene and the BGHpA. This plasmid was named pEC120CpG. The eukaryotic expression units from pEC120 and pEC120CpG were cloned into the *L. lactis *pJAG5 cloning vector [10]. PJAG5 contains the *hom*-*thrB *auxotroph marker, the minimal theta type replicon, and a polylinker region for cloning (Fig. 1). The threonine auxotrophic *L. lactis *strain, MG1614ΔhomthrB, was used for cloning and plasmid production [12]. Selection of primary transformants and growth under selective conditions was done in defined medium lacking threonine [10]. The HIV-1_BX08 _gp120 expression unit was obtained from pEC120 using PCR and primers homologous to the 5' end of the CMV promoter (5'-CGGGGTACCCTTGTGCAATGTAACATCAGAG-3') and to the 3' end of the BGHpA (5'-CGGGGTACCCTGTGGATAACCGTATTACCG-3'). Similarly, we isolated the gp120 expression cassette including the 80 bp CpG motif using PCR, the same primers and pEC120CpG as template DNA. The primers introduced terminal *Kpn*I restriction sequences (underlined). The gp120 expression units were ligated to *Kpn*I-treated pJAG5 and electroporated into competent *L. lactis *MG1614ΔhomthrB. PCR and DNA sequencing confirmed the correct structure of the chimeric pJAG5 vectors. The resulting 7.8 kb vaccine plasmids were named pLL120 and pLL120CpG.

### Plasmid preparation for DNA vaccine production

The *E. coli *based plasmids were produced and purified using Qiagen Midiprep according to the manufacturer (Qiagen, Hilden, DE). The *L. lactis*-based plasmids were purified similarly but with modifications as follows. A 100 ml (OD_600 _= 3) culture was harvested and washed in 20 ml TE buffer containing 7% sucrose (STE). Cells were resuspended in 4 ml STE containing 20 mg/ml lysozyme (Sigma) and 0.1 mg/ml RNase H (Sigma) and incubated at 37°C for 60 min. Four ml NaOH-SDS buffer (#P2 from Qiagen) was added and incubated for 5 min at room temperature. Four ml of ice cold potassium acetate buffer (#P3 from Qiagen) was added and mixed before incubated 30 min on ice and centrifuged at 20,000 *g *at 4°C for 1 h. The clear supernatant was applied to midi cartridges and thereafter handled as suggested (Qiagen). The quality of the PBS dissolved DNA was analysed by agarose gel electrophoresis and evaluation of the A260/A280 ratio.

### *In vitro *expression of gp120_BX08 _protein

To examine eukaryotic expression the syn.gp120_BX08 _gene, the human HEK293 kidney fibroblast cell line (ATCC, Rockville, MD) was transfected with pLL120, pLL120CpG, pEC120, and pEC120CpG, respectively, using Effectene transfection kit (Qiagen). Radioimmunoprecipitation (RIPA) of ^35^S-met and ^35^S-cys labelled protein using gp120 specific antibodies was done as described [5].

### DNA vaccination

6–7 weeks-old BALB/c mice were purchased from Bomholdtgaard (Taconic, Ry, DK) and kept in groups of five per cage with food and water *ad libitum *and artificial light 12 h per day. The acclimatization period before immunization was 5 days. Mice were immunized intramuscularly (i.m.) in *Tibialis anterior *with 50 μl of 2 mg/ml plasmid in endotoxin-free PBS buffer using pLL120, pLL120CpG, pEC120, and pEC120CpG, respectively. The four vaccine constructs were tested on four groups of 10 mice each (n = 10). A group of 5 mice received PBS buffer alone and served as a negative control. Mice were vaccinated at week 0, 9 and 15. Blood samples were drawn one day prior to the first immunization as a pre-immune control serum (day 0), and thereafter every third week. The experiment was terminated at week 19.

### Serological assays

Mouse anti HIV-1 gp120 antibodies were measured by indirect ELISA. Wells of polystyrene plates (Maxisorb, Nunc, Roskilde, DK) were coated for 2 days at room temperature with HIV-1 IIIB recombinant gp120 (Intracel) at 0.2 μg/100 μl of carbonate buffer, pH 9.6. Before use the plates were blocked for 1 h at room temperature with 150 μl/well of washing buffer (PBS, 0.5 M NaCl, 1% Triton-X-100) plus 2% BSA and 2% skim milk powder. After 3 × 1 min washings, mouse plasma was added at 100 μl/well diluted in blocking buffer and the ELISA plates were incubated for 90 min at room temperature using a microtiter plate shaker. The standard curve was made from a pool of plasma consisting of BX08 gp120 positive mice sera [5]. Plates were again washed 5 × 1 min and incubated 1 h at room temperature with 100 μl/well of HRP-conjugated rabbit anti-mouse IgG (DAKO-Cytomation, Glostrup, DK) diluted 1:1000 in blocking buffer. Colour was developed with 100 μl/well of peroxidase enzyme substrate consisting of 4 mg of *o*-phenylenediamine in 11 ml water plus 4 μl hydrogen peroxide (30%, w/w). The reaction was terminated after 30 min by 150 μl/well of 1 M H_2_SO_4_. The optical density (OD) of wells was measured at 492 nm using a microplate photometer. Anti-HIV-gp120 IgG titers were expressed as the reciprocal of the plasma dilution resulting in an OD_492 nm _value of 0.500.

IgG1 and IgG2a were analysed by capture gp120 sandwich ELISA [24,25]. Briefly, Maxisorb 96-well plates (NUNC) were pre-coated overnight with PBS containing 1 μg/ml sheep-anti-gp120 (Aalto, Dublin, IR), washed 5 times in PBS containing 0.05% Tween-20 and incubated for 2 h at room temperature with 100 μl 1% Triton X-100 treated cell-free culture supernatant from 5.25.EGFP.Luc.M7 cells (donated by Ned Landau, Salk Institute, CA, USA) which were infected chronically with HIV-1_BX08_. A dilution series of plasma samples from each mouse was incubated for 2 h at room temperature. Wells were washed five times in PBS-Tween-20 and incubated with HRP-conjugated Rat anti-mouse IgG1 or -IgG2a antibodies (Pharmingen Brøndby, DK) both diluted 1:1000 in dilution buffer (0.5 M NaCl, 3 mM KCl, 15 mM KH_2_PO_4_, 6 mM Na_2_HPO_4_:2H_2_O, 2% Triton-X 100, 1% w/v BSA, 0.1% w/v phenol-red) and incubated for 1 h at room temperature. Wells were washed five times as above and once using distilled water before colorimetric development using 100 μl *o*-phenylenediamine substrate solution (Kem-En-Tec, Taastrup, DK). The reaction was stopped with 150 μl 1 M H_2_SO_4 _and absorbance measured at 490 nm.

### Intracellular IFN-γ cytokine analysis by flow-cytometry (IC-FACS)

Mouse spleens were removed aseptically at week 19 and gently homogenized to single cell suspension, washed 3 times in cold RPMI1640 (Invitrogen, Taastrup, DK) supplemented with 10% fetal calf serum (FCS) (Invitrogen) and resuspended to a final concentration of 5 × 10^7 ^cell/ml. Spleens from individual mice were homogenised and transferred to a round-bottom 96-well plate (2 × 10^5 ^cells per well). Wells, containing a total volume of 200 μl RPMI1640 with 10% FCS, were incubated for 6 h at 37°C with 50 U/ml of IL-2 (Roche, Hvidovre, DK), monensin (3 μM) (Sigma, Brøndby, DK) with and without a 15-mer peptide derived from the V3-loop of HIV-1_BX08 _containing a conserved murine H-2D^d ^restricted CTL epitope (IGPGRAFYTT). Wells were washed and cells re-suspended in 50 μl medium containing fluorescent antibodies for the relevant surface markers, CD4-FITC, CD8-PECy7, and CD44-APC (Pharmingen) and incubated for 20 min in the dark at 4°C. Then 100 μl monensin (3 μM) in PBS was added before centrifugation at 400 × g for 5 min. Cells were resuspended in 100 μl monensin (3 μM) in PBS and 100 μl 2% paraformaldehyde (Merck, Damstadt, DE) and incubated for 30 min in the dark at 4°C. Cells were pelleted and resuspended in 100 μl PBS solution containing 3 μM monensin and 1% BSA. Cells were pelleted and resuspended in 200 μl PBS containing 0.5 % Saponin (Sigma) and incubated in the dark for 10 min at room temperature. Cells were pelleted and incubated for 20 min at 4 °C with 50 μl 0.5 % saponin solution containing PE conjugated anti-IFN-γ antibodies (Pharmingen) to stain for intracellular IFN-γ. Cells were washed twice with 100 μl 0.5 % saponin/PBS solution before addition of a fixing solution containing 2% paraformaldehyde. Fixed cells were analysed on a BD LSR-II flowcytometer (Becton Dickenson) using the BD FACS Diva software.

### Cytotoxic T lymphocyte assay

For assay of cytotoxic T lymphocytes (CTL) the splenocytes were incubated 5 days with mitomycin-C treated (50 μg/ml for 1 h) and CTL epitope peptide loaded mouse P815 (H-2D^d^) stimulator cells at a ratio of 10:1 in medium supplemented with 5 × 10^-5 ^M β-mercaptoethanol. For assay of cytolytic CTL response to HIV-1_BX08_, P815 target cells were pulsed with 20 μg/ml of the HIV-1_BX08 _V3 peptide SIHIGPGRAFYTTGD containing the H-2D^d ^restricted CTL epitopeCorbet, 2. After stimulation splenocytes were washed three times with RPMI1640 supplemented with 10% FCS and resuspended to a final concentration of 5 × 10^6 ^cells/ml. Cell suspension (100 μl) was added in triplicate to U-bottom 96-well microtiter plates and a standard 4 h ^51^Cr-release assay performed as described [4].

### *In vitro *splenocyt activation by plasmid DNA

Splenocytes were prepared from spleens of non-immunized 6–12 weeks old female BALB/c mice. Cells were cultured in 96-well U-bottom plates at 37°C and 5% CO_2 _and maintained in RPMI1640 supplemented with 10% FCS, 1% penicillin/streptomycin, and 0.1% mercaptoethanol. Splenocytes (3 × 10^5 ^cells/well) were treated with RPMI1640 medium, plasmid (3 ug/well) or plasmid DNA treated with DNase I (Biolab, Risskov, DK) using 1 U DNase per 1 ug DNA for 1 hr at 37°C followed by inactivation at 75°C for 10 min. The synthesis of IL-6 and IFN-γ in supernatants of cells cultured for 0, 1, 2, and 3 days was measured by sandwich ELISA kits (R&D Systems, Minneapolis, MN).

## Results

### Vectors and immune stimulatory CpG motifs

The characteristics of the *L. lactis *(pLL120 and pLL120CpG) and *E. coli *(pEC120 and pEC120CpG) based vectors are shown in Fig. 1. Sequence analysis showed that the *L. lactis *vector backbone had a lower GC content (37%) than the *E. coli *vector (48%) and contained two copies of a known immune stimulatory mouse CpG motifs (GA**CG**TT), while the *E. coli *vector backbone only contained one CpG motif. Even though the synthetic gp120 gene is very GC rich (56%) because of the humanized codons, it did not contain classical ISS motifs, but did harbour three human CpG motifs (two copies of GT**CG**TT and one copy of CT**CG**AG). The added CpG minigene contained 12 CpG dinucleotides giving rise to six consensus ISS CpG motifs (5'-two purines-CpG-two pyrimidines-3') including two copies of the mouse ISS CpG motifs (GA**CG**TT and AA**CG**TT) [22]. Thus, when inserted the CpG minigene increased the numbers of mouse ISS motifs by two. However, unidentified mouse ISS or immune inhibitory CpG motifs may be present in the plasmids.

### Eukaryotic gp120_BX08 _expression using a *L. lactis *based vector

**Figure 2:**
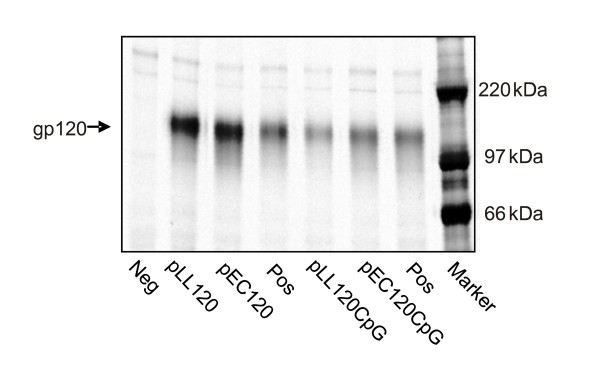
**Expression analysis of gp120**. *In vitro *expression of gp120 protein from *L. lactis *(pLL120) and *E. coli *(pEC120) plasmids with and without CpG motifs. RIPA of culture supernatants of ^35^S-cys/met-pulsed HEK-293 cells transfected with the vaccine plasmids. Precipitation is done using pooled serum from HIV positive patients and the immunoprecipitates were analysed by SDS-PAGE. Positive control plasmid (Pos), untransfected cells (Neg), molecular marker (Marker). The gp120 is indicated.

To evaluate the influence of the plasmid backbone on the expression levels of the gp120 gene, human kidney cells were transiently transfected. The expression of the syn.gp120_BX08 _gene using the *L. lactis *or *E. coli*-based vectors was examined by RIPA of ^35^S-labelled supernatant proteins from transfected HEK293 cells and purified IgG from a pool of HIV sero-positive patients. ^35^S-labelled supernatant from untransfected cells served as a negative control and was also precipitated but did not produce a protein with a molecular weight of 120 kDa (Fig. 2). Cells transfected with the any of the four vaccine plasmids produced a 120 kDa product (Fig. 2). The expression levels of gp120 were comparable for pLL120 and pEC120 as well as for pLL120CpG and pEC120CpG. For both types of vectors the amounts of gp120 expressed seemed somewhat lower when they harboured the CpG cassette. The *L. lactis *based vectors drive therefore antigen synthesis as efficiently as the *E. coli *based vectors. Furthermore, the produced gp120 was immunogenic as antibodies from HIV-1 sero positive patients recognize the *in vitro *produced HIV protein (Fig. 2).

### Antibody response after genetic immunization

**Figure 3:**
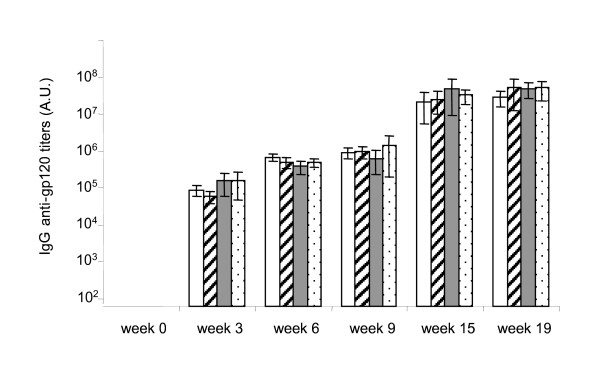
**Antibody response after DNA vaccination**. Mouse IgG anti-gp120 antibody titers at week 0, 3, 6, 9, 15, and 18. DNA immunizations were at week 0, 9, and 15. The antibody titer of individual mice was determined and presented as the mean of the group of 10 mice (n = 10). Error-bars represent +/- two times Standard Error of Mean (2 × SEM = SD/√n). White bars:pLL120CpG, cross-sectioned bars: pLL120, grey bars: pEC120, dotted bars: pEC120CpG.

We examined the humoral responses in the DNA vaccinated mice using ELISA (Fig. 3). The four different plasmids (Fig. 1) were each administered to four groups of 10 mice, respectively. Mice immunized i.m. with the pLL120 and pLL120CpG at weeks 0, 9, and 15 induced specific IgG antibodies to the gp120 protein with the same kinetics and maximum titers as the mice receiving the *E. coli *based plasmids. The first immunization induced high specific antibodies in all mice. The titers obtained were similar to those obtained in previous mouse DNA vaccination experiments using the syn.gp120_BX08 _in WRG7079 [5]. The second immunization at week nine boosted the antibody titers about one hundred times in all groups. Further boosting at week 15 had less effect. The titers obtained at each time point were indistinguishable from those obtained with the corresponding vectors containing the putative ISS CpG cassette. The negative control group receiving buffer alone showed no anti-gp120 antibody titers above the background (data not shown). Thus, the kinetics and the magnitude of the anti-gp120 antibody induction was similar using the *L. lactis*-based pJAG5 and the *E. coli*-based plasmid backbones.

**Figure 4:**
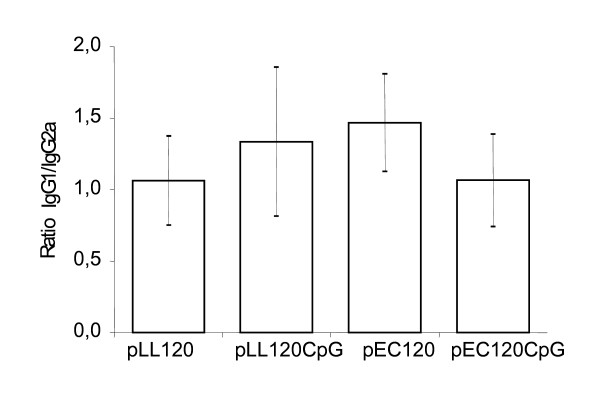
**Antibody subtypes following immunization**. Ratio IgG1/IgG2a anti-gp120 antibodies from samples of week 18. Antibody subtypes of individual mice was determined and a mean of each group (n = 10) is shown with error-bars representing 2 × SEM.

The antibody subclass profile of the induced response was analysed. Here gp120 specific IgG1 and IgG2a were determined by ELISA to be used as a surrogate marker for the Th1/Th2 balance (Fig. 4). Mice immunized with the *L. lactis *based plasmids induced an IgG1/IgG2a ratio indistinguishable from that induced by the *E. coli *based pEC120 and pEC120CpG. Furthermore, the IgG1/IgG2a ratios obtained from the data from similar vectors with and without the CpG cassette were statistically indistinguishable (two sample t-test, P > 0.05). All IgG1 and IgG2a ratios were above 1 and below 1.5, which is indicative of a mixed Th1:Th2 response with a slight Th2 predominance (Fig. 4).

### CD8^+ ^T lymphocyte immune responses after genetic immunization

**Figure 5:**
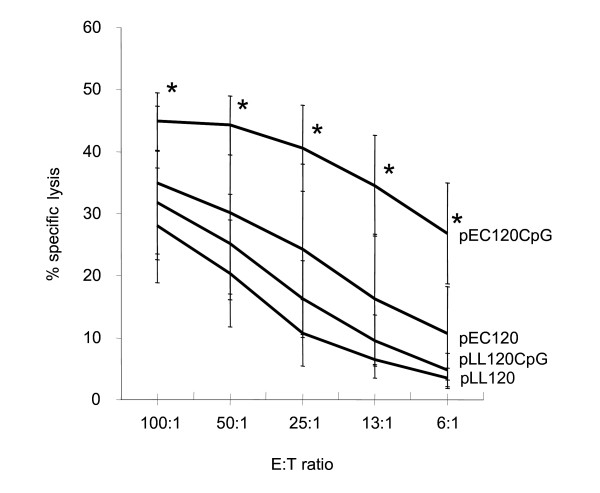
**Cellular immune response after DNA immunization**. Peptide-specific CTL responses were determined by ^51^Cr-release assay at titrated levels of effector to target cell (E:T) ratios. Splenocytes or effector cells were pre-stimulated for five days *in vitro *with V3-loop peptide. Assay is done on individual mice and presented as a mean of each group +/- 2 × SEM, n = 10. Significant difference between pEC120CpG and pLL120CpG is indicated with asterisks () (two sample t-test, P < 0.05).

**Figure 6:**
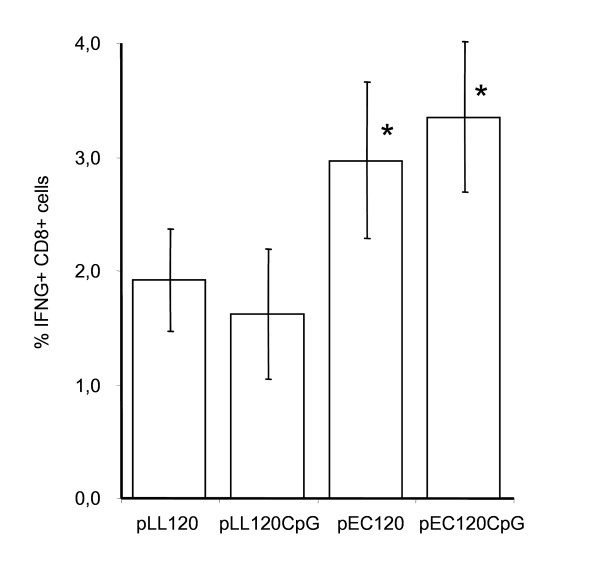
**Peptide-specific IFN-γ induction**. CD8^+ ^splenocytes of immunised mice was determined by flow-cytometry of samples taken at week 18. Error-bars represent +/- 2 × SEM. Asteriks () indicate significant differences (two sample t-test, P < 0.05) between pEC120 and pLL120 and between pEC120CpG and pLL120CpG.

We examined the cellular immune response in mice after genetic immunizations with syn.gp120_BX08 _by cytolytic CTL assay and staining for intracellular induction of IFN-γ (IC-FACS) (Figs. 5 and 6). A strong cellular immune response was induced by the four vectors that were significantly higher than both the background (unloaded target cells) and the flat response obtained from unimmunized mice (data not shown). This confirms the *in vivo *expression and immunogenicity of gp120_BX08 _expressed from both *L. lactis *and *E. coli *vectors. After five days of epitope stimulation a somewhat higher cytolytic CTL response was obtained with the *E. coli*-based plasmid as compared to the *L. lactis-*based plasmid (Fig. 5). Addition of the ISS CpG cassette increased this specific CTL response for both vectors albeit more pronounced for the *E. coli *based vector (Fig. 5).

The cellular response was also analysed by IC-FACS staining for direct epitope-peptide-induced IFN-γ production in CD8^+ ^T-cells (Fig. 6). The analysis confirmed specific cellular immunity induced by the vaccine gene in the four vectors. This IFN-γ induction was, however, lower for the *L. lactis *based vectors than for the corresponding *E. coli*-based vectors.

### *In vitro *stimulation of splenocytes by plasmid DNA

**Figure 7:**
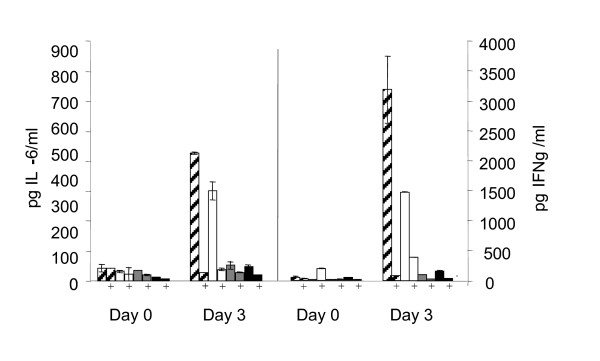
**Non-specific immune stimulatory effect of vaccine plasmids**. Splenocyte supernatants were collected before stimulation (day 0) and three days post stimulation with the plasmids (day 3). The secretion of IL-6 and IFN-γ was analysed by ELISA. Cross-sectioned bars: pLL120CpG, white bars: pLL120, grey bars: pEC120, black bars: pEC120CpG, and bars with vertical lines: culture medium. Symbols: +: DNase treatment of plasmid preparation. Error bars represent SD of two independent experiments.

To evaluate the immune stimulatory properties of the vaccine plasmids splenocytes were co-incubated with plasmid DNA and the induction of pro-inflammatory cytokines were analysed. Interestingly, the *L. lactis-*based vectors induced a higher synthesis of both IFN-γ and IL-6 than the corresponding *E. coli *vectors (Fig. 7). Furthermore, addition of the CpG minigene to the *L. lactis *vector caused a significantly higher induction of both IFN-γ and IL-6 compared to the pLL120 without the CpG minigene. To analyse if this stimulatory effect is caused by the plasmid DNA or by contaminants in the DNA preparation the DNA was enzymatically degraded. DNase treatment of the plasmid solutions abolished the pro-inflammatory effect (Fig. 7). Therefore, the immune stimulatory induction by the *L. lactis *vaccine vectors is directly linked to the plasmid DNA and not to contaminants that may be present in the DNA preparations.

## Discussion

Plasmid DNA vaccines are the next generation of vaccines. As yet, research has mostly been focused on building functional vaccines by increasing the antigenicity of the encoded gene or by new plasmid delivery methods. Therefore, focus on the plasmid backbone and on the microbial production host has been limited. The key driver of this study was to develop an alternative plasmid backbone in DNA vaccines and a new microbial production host and compare it to a traditional used *E. coli *based DNA vaccine. This study represents to our knowledge the first alternative to *E. coli *based plasmid DNA vaccines and provides new information on the role of the plasmid backbone in DNA vaccines.

We examined the use of a novel *L. lactis *vector as the plasmid backbone in DNA vaccines. The main advantages of this vector and its production strain are avoidance of antibiotic resistance genes and antibiotic contaminants, lack of LPS and endotoxins, and improved safety as the *L. lactis *system may be regarded as food grade. In the presence of the *thr*-carrying plasmid backbone, the auxotrophic host strain is able to grow in a chemical defined medium in the absence of threonine. The system is therefore compatible with use in a growth medium free of animal components that may contain viruses and prions. Specifically, we constructed *L. lactis*-based expression vectors with and without an ISS CpG unit containing the HIV-1_BX08 _gp120 vaccine gene with humanized codons and developed a suitable process for purification of plasmid DNA from *L. lactis*. Furthermore, we demonstrated eukaryotic expression *in vitro*, and DNA immunizations of mice and compared the resulting humoral and cellular immune responses with those obtained from induction with traditional *E. coli-*based vectors [5].

Plasmid DNA purification from Gram-positive bacteria is complicated due to the presence of a thick peptidoglycan cell wall. Purification of *L. lactis *plasmid DNA has relied on modified alkaline procedures [26] including the use of toxic substances like phenol, chloroform and ethidium bromide. The method presented here conveniently employs the commercial ion exchange cartridges with a few modifications including an initial treatment using a cell wall degrading enzyme. The developed plasmid preparation contained only low amounts of genomic DNA and RNA and was proven useful in routine analysis of plasmids, transformation, *in vitro *transfection of human 293 cells, and *in vivo *DNA immunization. Although *L. lactis *is attractive in terms of safety the efficiency of the system is a drawback. Due to the medium copy number of the pJAG5 plasmid the yield during a fermentation process is 5 fold lower than that of high copy number plasmids from *E. coli *(data not shown). However, higher copy number plasmids were also tested in *L. lactis *but showed lower segregational stability or a less favourable distribution between supercoiled and non-supercoiled plasmid forms (data not shown).

The transfection of human HEK293 cells demonstrated HIV-1_BX08 _gp120 expression using the *L. lactis *pJAG5 backbone as delivery vehicle. The difference in nucleotide content and/or difference in methylation pattern in *L. lactis *DNA could potentially influence the expression and/or the immune response. However, the intensity in RIPA of gp120 produced from cells transfected with pLL120 and pLL120CpG was similar to that of cells transfected with *E. coli *vectors. This illustrates that the backbone vector did not affect the level of expression, which may not be surprising since both vectors contain the same gp120 expression unit. The reasons for a somewhat lower *in vitro *expression from both vectors when the ISS CpG unit is included are not known but could be partly due to DNA methylation and gene silencing [27,28].

The usefulness of pJAG5 to carry the eukaryotic gp120 expression unit and induce an antibody response to the encoded gp120 was evaluated by naked DNA immunization. Mice serum showed IgG antibodies specific for gp120 and the induction followed the same kinetics and potency as that obtained using the corresponding *E. coli *plasmids. Moreover the IgG1/IgG2a ratios obtained with the *L. lactis *and the *E. coli*-based vectors were not statistically different in this mouse model. Thus, the induced Th1/Th2 balance determined by the IgG1/IgG2a surrogate marker was similar for the *L. lactis *and *E. coli *based-vector, respectively. Interestingly, both antibody titers and IgG1/IgG2a ratios were largely unaffected by the introduced CpG minigene supposed to act as a Th1 adjuvant via the Toll-like receptor 9 [29-33]. It cannot be ruled out that the use of a different and less Th2-prone mouse strain or higher doses of plasmid DNA could give minor differences. Some investigators have also observed minimal effect on the humoral immune response induced by DNA vaccine plasmids with different numbers of CpG motifs present [34,35]. Thus, as opposed to the established immune stimulatory effect of synthetic and single-stranded oligodeoxynucleotides, the effect of plasmid DNA is more controversial.

In contrast to the identical humoral response induced by the four plasmids, CD8^+ ^T-cells were activated less by the *L. lactis*-based vectors than by the *E. coli*-based vectors as measured by both IC-FACS and CTL activity. In previous studies of synthetic HIV-1 envelope genes from strains BX08 and MN no correlation was found between the CTL response and the expression levels, secreted or membrane-bound antigens, mode of DNA delivery, T helper cell response, or antibody response [5,36,37]. This may not be surprising since the mechanisms and constraints involved are quite different. The similar humoral but lower cellular immune response from the *L. lactis*-based vector could be due to a lower adjuvant effect on CD8^+ ^T cells exerted by differences in ISS CpG motifs since the introduction of the CpG unit showed a tendency to increase the CTL activities within each group of mice (Fig. 5). Various CpG ISS's, their numbers, location, or their spacing or the sequences flanking them may stimulate the immunological active cells and their cytokine productions differently, and in this way differentially influence the resulting immune responses [35]. Both the presence of unknown CTL immune-neutralising sequences in the plasmid backbone [17,38] or lack of contaminating LPS can cause the lower CTL induction observed using the *L. lactis *based DNA vaccine. To explain this we evaluated the immune activating capacity of the plasmids on splenocytes. Surprisingly, the *L. lactis *plasmids showed higher *in vitro *immune stimulatory properties than the tested *E. coli *plasmids. To test if this pro-inflammatory effect was due to contaminating components like LPS from *E. coli *or teichoic acid from *L. lactis*, the plasmid was enzymatically degraded. The results indicate that the inflammatory stimulation of the splenocytes was due to the plasmid DNA itself. As such the *in vitro *stimulation assay cannot explain the lower cytotoxic T-cell response induced by the two *L. lactis *vectors. Furthermore, the higher adjuvant effect of the *L. lactis *vectors *in vitro *does not correlate with the DNA immunizations *in vivo *where the two systems induce similar antibody responses. This emphasizes the importance of animal studies. Because of the complex nature of the adjuvant effect of plasmid DNA, we measured the resulting effect of *in vivo *DNA vaccination using large amounts of DNA as routinely used in i.m. immunization without any pre-treatment or added adjuvant to allow for maximal immune interference of the plasmids. The observed difference in the cellular but not humoral immune induction by the *L. lactis *and *E. coli*-based vectors may lead to new ways to adjuvant DNA vaccines. Here, the *L. lactis *vector can be a tool to analyse DNA sequences that can be added for maximum induction of cellular immune responses. The development of an improved *L. lactis *based plasmid that can induce a stronger cellular immune response is however essential for this to be a true alternative to *E. coli *based DNA vaccines.

## Conclusion

By this study we suggest an alternative host vector system for use as plasmid DNA vaccines. The *L. lactis *system is based on an endotoxin-free and safe organism and the plasmid backbone is free of antibiotic resistance genes. The *L. lactis *based DNA vaccine vector expressed HIV-1_BX08 _gp120 vaccine component similarly to *E. coli *vectors and induced similar humoral immune responses as corresponding *E. coli *based plasmids. However, compared to *E. coli *plasmids the *L. lactis *based DNA vaccine system induced a lower cellular immune. This is not due to lack of immune stimulatory effect as the *L. lactis *based plasmid showed high pro inflammatory effect on splenocytes. Although the developed system is less potent compared to the tested *E. coli *system it may be a valuable tool to identify new CTL adjuvant components.

## Competing interests

The author(s) declare that they have no competing interests.

## Authors' contributions

GJG carried out the immunological studies together with JG. MT made the *in vitro *splenocyte assay testing the immune stimulatory role of the plasmids. AFO supervised the immunological studies, the design of the vaccine plasmids and helped to draft the manuscript. SMM gave helpful input to the genetic design of the plasmids. JG made the DNA vaccine constructions, vaccine production and drafted the manuscript.
